# The pleasure of effort: Cognitive challenges trigger hedonic physiological responses

**DOI:** 10.1111/nyas.15323

**Published:** 2025-03-24

**Authors:** Jakub Kraus, Christopher Mlynski, Franziska Hartmann, Georgia Clay, Thomas Goschke, Giorgia Silani, Veronika Job

**Affiliations:** ^1^ Department of Occupational, Economic and Social Psychology, Faculty of Psychology University of Vienna Vienna Austria; ^2^ Institute of General Psychology, Biopsychology and Methods of Psychology, Faculty of Psychology TU Dresden Dresden Germany; ^3^ Department of Clinical and Health Psychology, Faculty of Psychology University of Vienna Vienna Austria

**Keywords:** effort, facial EMG, learned industriousness, liking, reward

## Abstract

Challenging prominent neuroscientific conceptions of effort as generally aversive, recent research suggests that people can learn to seek effort. Importantly, it is unknown whether people once they learn to value effort for its instrumentality, experience pleasure when engaging in effortful tasks. In this preregistered study (*N* = 194), we tested the hypothesis that effort‐contingent rewards in a cognitive task will induce reward‐related hedonic facial responses before, during, or after effortful engagement in a subsequent non‐incentivized task. The results showed that effort‐contingent reward enhanced participants’ facial responses in the zygomaticus major (ZM) muscle after effort exertion (consumption phase) in the subsequent non‐incentivized task, especially in high‐difficulty trials. Electrical activity in the ZM was positively associated with subjective pleasure ratings in the experimental group when solving difficult trials, suggesting that it is implicitly tracking the hedonic value of effort. Our findings show that effort‐contingent reward promotes effort‐related reward experience, indicating that effort itself becomes intrinsically rewarding as experienced pleasure after effort exertion.

## Graphical abstract

Effort is thought to be generally aversive but we tested the hypothesis that effort‐contingent rewards in a cognitive task will induce reward‐related hedonic facial responses in a subsequent non‐incentivized task. The results showed that effort‐contingent reward enhanced participants’ facial responses in the zygomaticus major (ZM) muscle after effort exertion (consumption phase) in the subsequent non‐incentivized task, especially in high‐difficulty trials. Such responses were further positively associated with their pleasure ratings.

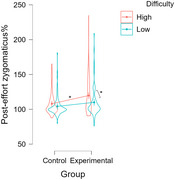

## INTRODUCTION

Effort is generally seen as aversive,^1^, ^2^ as something people avoid whenever possible.^2^ However, recent evidence from laboratory and online experiments suggests that people can learn to value effort positively and approach it even if this does not lead to immediate extrinsic benefits.^3^, ^4^ The basic principle is grounded in learning mechanisms of conditioning, which suggest that effort can become a secondary reinforcer if it is repeatedly followed by a reward, thereby increasing its inherent value and individuals’ willingness to approach effortful situations. The resulting acquired tendency to seek and exert effort has been called learned industriousness.^5^ It was first documented in studies showing that rats or children persisted longer and worked harder on a task after they had been rewarded for performing a hard as compared to an easy task.^6^, ^7^

Recently, experimental paradigms have been developed to demonstrate that rewards paired with effort, independently from performance, promote successive effort approach behavior. The novel feature in the respective studies was that reward was not made contingent on performance, but on a physiological measure of effort, which allowed directly rewarding effort rather than performance. To this end, cardiovascular (CV) indicators of effort exertion were assessed while participants completed a series of working memory tasks varying in task difficulty.^3^ Participants in the experimental group received a reward that was contingent on the effort they exerted during task engagement, while participants in the control group received random rewards that were neither contingent on the exerted effort nor on task performance. On a subsequent math task, in which participants were informed that they would no longer receive any extrinsic reward, participants in the experimental group chose to work on more demanding tasks as compared to the control group. Similar (albeit weaker) results were obtained in a study where effort‐contingent reward was compared to rewards based on performance.^4^

Taken together, previous studies document that people can learn to approach effort and choose more demanding tasks even if the exerted effort does not result in extrinsic reward. This result is consistent with the idea that effort gained an intrinsic value, having become a secondary reinforcer. However, as of yet, it is unknown whether participants just learn to value effort as being instrumental to obtain a possible reward, or whether effort itself receives rewarding qualities and is accompanied by the experience of pleasure. The present research aimed to assess reward processing in participants who had completed a learned industriousness treatment^3^ to test whether they would show signs of effort‐related experience of pleasure. Participants in this study first completed a training phase during where they engaged in a series of working memory tasks varying in difficulty. The obtained reward after each block was either based on their CV reactivity (heart pre‐ejection period, PEP) or, in the control condition, yoked to a previous participant in the experimental group. In the following transfer phase, all participants completed high‐ or low‐difficulty math tasks. During the math task, effort‐related reward experience was assessed with facial muscle reactions to objectively track implicit hedonic responses,^8^, ^9^, ^10^, ^11^, ^12^, ^13^ as well as with subjective ratings of wanting and liking.

Ample animal and human research distinguishes between two distinct phases of reward processing.^13^, ^14^, ^15^ First, reward anticipation includes processes that underlie incentive salience (i.e., the mesocorticolimbic dopamine‐mediated desire for rewards and the motivation to implement instrumental behavior to gain them). Second, reward consumption encompasses the pleasure of consuming the reward, which is (mainly) mediated by opioid‐related processes in hedonic hotspots in cortical and subcortical brain regions.^14^ In the present research, we applied this distinction to the notion of a possible intrinsic value of effort, which might likewise be processed in different phases implying distinct processes: (a) during effort anticipation as anticipatory pleasure and the motivation aspect pushing toward effort exertion (“wanting” to exert effort) and (b) during/after effort exertion as consummatory pleasure, that is, the hedonic emotional response to exerting effort itself (“liking” of exerting effort). Specifically, the objective measures of the hedonic and motivational aspects of the intrinsic value of effort were obtained by measuring the activity of the muscles zygomaticus major (ZM) (involved in smiling) and corrugator supercilii (involved in frowning) via facial electromyography (fEMG) recording.^13^ The fEMG assessment was further coupled with trial‐by‐trial self‐reports to characterize which aspect of reward processing was driving changes in the muscle activity, along with the continuous CV measurement of the heart PEP.

Based on behavioral research indicating increased value of effort through effort‐contingent reinforcement^3^, ^4^, ^6^ and evidence of fEMG to reflect reward processing,^9^, ^10^, ^11^, ^12^, ^13^ we formulated four preregistered hypotheses. First, we hypothesized that providing effort‐contingent reward, as compared to effort‐noncontingent reward in the learning task, will (a) increase the ZM and/or (b) decrease the corrugator supercilii electrical muscle activity before effortful task engagement on the transfer task, and/or during/after effortful task engagement on the transfer task. Such changes in muscle activity should especially be present during the hard trials where more effort is required. For the subjective ratings, we hypothesized that providing effort‐contingent reward, as compared to effort‐noncontingent reward in the learning task, will increase the subjective (a) wanting and (b) liking of the transfer task. Any resulting group differences before task engagement on the transfer task would reflect a hedonic response before reward obtainment (i.e., anticipatory pleasure). On the other hand, group differences during/after task engagement on the transfer task would support the involvement of the hedonic aspect of the intrinsic value of effort upon reward obtainment (i.e., consummatory pleasure). In case the group differences occur in both phases (i.e., before and during/after task engagement on the transfer task), it would imply that anticipatory as well as consummatory reward‐related processes were involved.

## METHODS

The study was preregistered (https://aspredicted.org/FWR_HH9), and the data, materials, and analyses code are available at OSF repository (https://doi.org/10.17605/OSF.IO/N2T3J).

### Participants

A total of 208 participants were initially enrolled in the study. Fourteen participants were excluded due to reasons determined through debriefing or data‐acquiring issues (*N* = 11/10% from the experimental and *N* = 3/3% from the control group). Five of these participants were excluded due to misunderstanding the instructions or the task (experimental = 3, control = 2). Five participants were excluded due to bad or lost contact of the CV electrodes (all from experimental). Four additional participants were excluded due to the task or data acquisition malfunction (experimental = 3, control = 1). The final sample thus resulted in *N* = 194 participants. Participants were divided randomly into either the experimental (effort‐contingent reward) group (*N* = 94) or control (effort‐noncontingent reward) group (*N* = 100). fEMG data of only one or both muscles were additionally excluded in another eight participants (experimental = 5, control = 3) due to insufficient electrode impedance. The accompanying logistic regression showed the presence of a differential attrition (discussed in Ref. 16) between the experimental and the control group (*χ* ^2^(1) = 5.04, *p* = 0.026). This result might be driven by the CV electrode malfunction that by chance happened only to the participants who were randomly assigned to the experimental group.

Ethical approval was obtained from the TU Dresden ethics committee and the study complied with the Declaration of Helsinki.^17^ All participants signed an informed consent form before participating and received a course credit or monetary compensation (as an 18‐euro base payment). Furthermore, all participants received an individually earned amount from the learning phase (see *N*‐back task section) of the experiment.

### The *N*‐back task (learning task)

**FIGURE 1:**
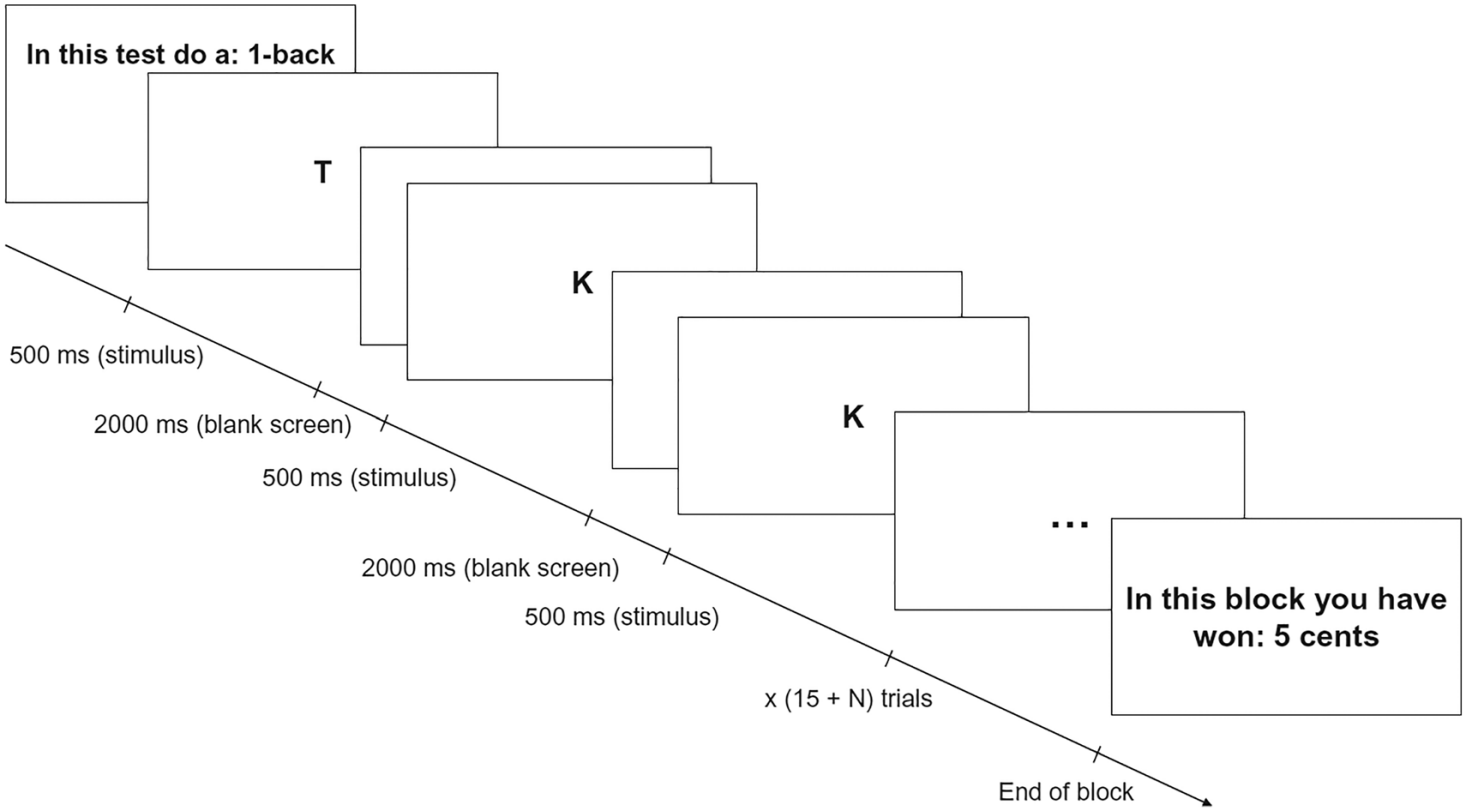
An illustration of a trial block in *N*‐back task. *Source*: Adapted from Ref. 3.

The *N*‐back task, a measure of working memory,^18^ was utilized to induce cognitive effort of varying intensities. Here participants were presented with long sequences of stimuli (in this case letters). For each item in the sequence, participants had to judge whether it matched the one presented n items ago, with n (the “load factor”) being manipulated between blocks of trials.^18^ There were four blocks of trials representing four levels of a “load factor” (“1‐back” to “4‐back”). First, participants completed practice trials, with one block at each level of difficulty. After each block they received performance feedback. In the main task, participants saw a sequence of letters and were instructed to determine whether the current letter was the same as the one presented *n* letters ago (i.e., “targets”). Participants had 2.5 s to press the “A” key if the letter was a target; no response was required for non‐targets. At the end of each trial, participants were informed that they will receive a monetary reward. This depended on the group they were assigned to (contingent/noncontingent reward) and the amount of effort exertion (for more detailed information, see the Procedure section). In total, there were 20 blocks consisting of five blocks of each 1‐, 2‐, 3‐, and 4‐backs in a randomized order (approximately 20 min). The typical *N*‐back trial is shown in Figure 1.

### Additional d‐prime measure

Responses to a previously seen stimulus at the *n*th position were a hit; not responding was a miss. Responses that were too early or too late were considered as a false alarm; not responding to an incorrect letter was a correct rejection. We calculated the d‐prime scores based on signal detection theory, that is, the proportion of (TotalHits—TotalFalseAlarms)/number of experimental blocks. The larger the d‐prime the better it reflects a participant´s working memory capacity (a potential indicator of subjective cognitive effort).^19^

### The math effort task (MET; transfer task)

**FIGURE 2:**
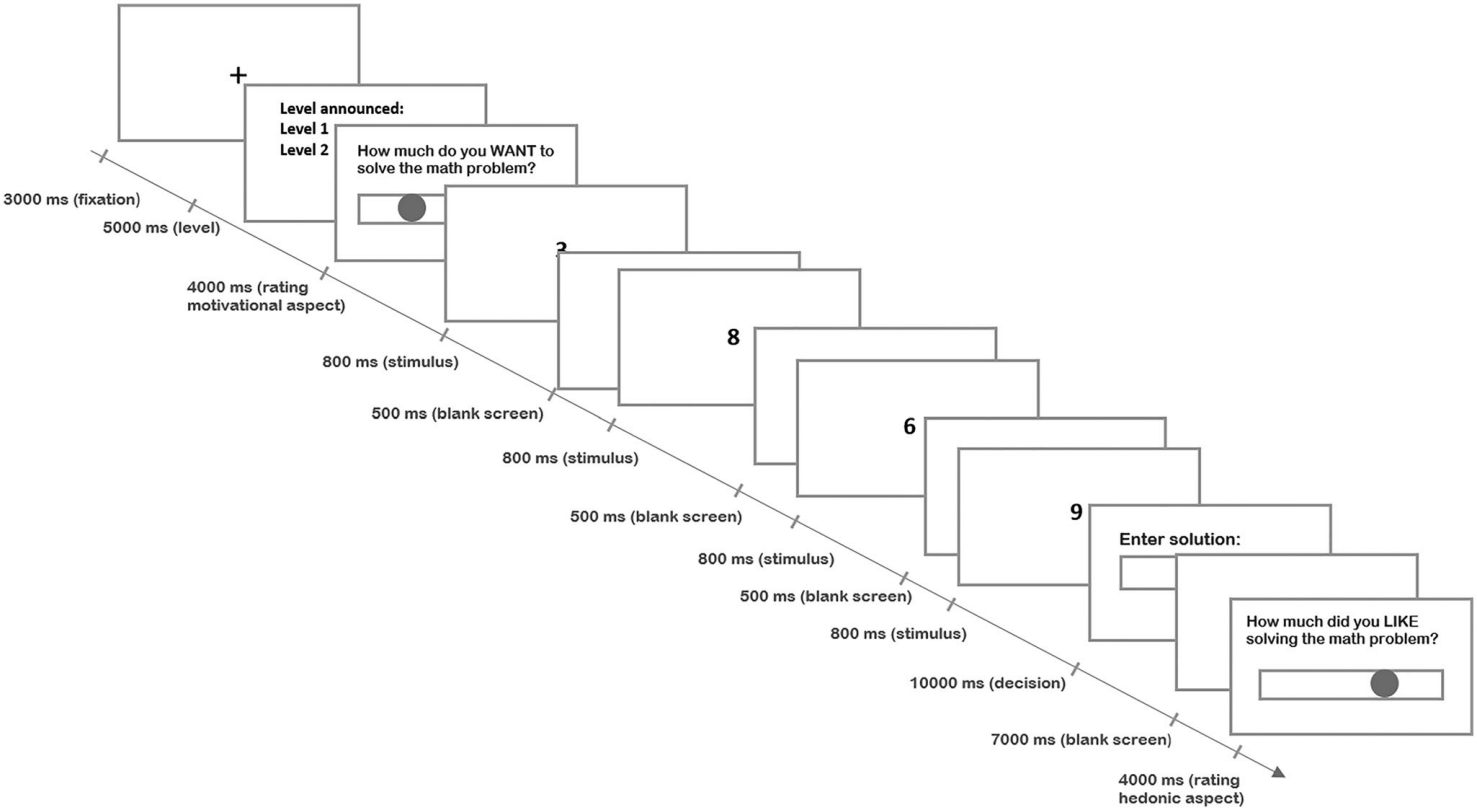
Illustration of the math effort task trial.

We used a modified version of the MET^20^ as the transfer task to assess the (neuro)physiological reward‐related activity (fEMG and CV reactivity) during exertion of effort. Participants were solving mathematical problems of various difficulty without receiving any extrinsic reward for task engagement. Here, participants worked through 40 trials of addition problems, each consisting of four numbers displayed one by one on the screen. At the beginning of each trial, there was a fixation cross for 3 seconds. Each of the numbers appeared in the middle of the computer screen for 0.8 s with an interstimulus interval (blank screen) of 0.5 seconds. Participants were instructed to add the four numbers within 10 s (task engagement phase). Once a problem was answered, a blank screen appeared for 7 s (post‐engagement phase). Next, participants rated the subjective pleasure (liking) elicited by solving the math problem on a continuous scale (no time restraint—approximately 4 s), after which they immediately proceeded to the next addition problem. The numbers in each trial were selected at random from a range that was determined by the level of difficulty. There were two levels of difficulty labeled as Levels 1 and 2. The specific level was announced at the beginning of each block of trials (*announcement*) and displayed for 5 seconds. The starting range for Level 1 included numbers from 1 to 9 and Level 2 included numbers from 7 to 35. In order to control for interindividual differences in math ability, there was a progressive calibration of the difficulty based on the previous performance of the participant. Specifically, if the accuracy on a Level 1 block was less than 80%, the highest two numbers of the range were removed. Similarly, for Level 2, if the accuracy was greater than 20%, the lowest two numbers of the range were removed. The task consisted of 20 trials of each difficulty level in blocks of 5 trials presented in a random order. Between every block, there was a 15 s break to allow CV activity to return to baseline. After each level announcement, participants rated their subjective wanting to solve math problems of a respective level (motivational aspect). In the MET, no feedback was provided to participants and participants were informed that the experimenter will not be able to see their performance. The typical MET trial is shown in Figure 2.

### Cardiovascular measurement (CV)

We noninvasively measured impedance cardiogram and electrocardiogram signals with a Cardioscreen 1000 system (Medis) to assess the heart PEP. PEP is the time interval between the onset of ventricular depolarization (Q point; i.e., beginning of electrical stimulation to the left ventricle) and cardiac ejection of blood from the heart (B point; i.e., the opening of the aortic valve). B‐point location was estimated based on the interval between the R peak of the electrocardiogram and the Z peak of the impedance cardiography dZ/dt wave form of valid heartbeat cycles.^21^ PEP (in milliseconds) was determined as the interval between R onset and B point.^22^ This is generally accepted as the gold standard for measuring beta‐adrenergic activation due to the lack of influence from changes in parasympathetic activity or vascular resistance, as is the case in other common beta‐adrenergic activation measures (e.g., systolic blood pressure and heart rate). Following precedent and recommendations by Llabre et al.,^23^ we operationalized CV response in terms of change scores (∆) computed by subtracting base values from values obtained in the learning and transfer phases. PEP ∆ obtained during the transfer phase was utilized for the purposes of our exploratory analyses. PEP ∆ obtained during the learning phase was used to ensure equal engagement with the *N*‐back tasks between both groups. PEP ∆ values served as an objective indicator of cognitive effort mobilization.^24^

### Facial electromyography (fEMG)

The reusable Ag/AgCl electrodes with 4 mm inner and 8 mm outer diameter were attached bipolarly based on the standard guidelines^25^ on the left corrugator supercilii (corrugator) and the ZM (zygomaticus) muscles. A ground electrode was attached to the participants’ forehead and a reference electrode on the left mastoid. The fEMG data were sampled at 1200 Hz with impedances below 20 kOHM using a MP160 data acquisition system (Biopac Systems, Inc.) and BioNomadix amplifiers (Biopac Systems, Inc.), and were recorded/(pre)processed in the AcqKnowledge software (version 5.0 Biopac Systems, Inc.). The fEMG was recorded during the entirety of the MET. The fEMG data were preprocessed with 20–400 Hz band‐pass filter and a 50 Hz notch filter, and rectified and smoothed with a 40 Hz low‐pass filter. Epochs were extracted focusing on the periods within: the intrinsic effort announcement (anticipation); effort exertion (task engagement); and the time after the exerted effort (post‐engagement) during the MET. In order to reduce the effect of non‐experimental movements on fEMG for each participant, epochs of raw data over the participant's mean ± 3 SD were removed. For each trial, values in the respective epochs were expressed as percentage of the average amplitude during the fixation cross at the beginning of that trial.

### Self‐reports and questionnaires

#### Ratings of wanting

The continuous visual analog scale ranged from “not at all” (left anchor) to “very much” (right anchor). Using the keyboard, participants were instructed to move the cursor along the continuum to express the degree of wanting to work on the announced mathematical problem. In each trial, the scale appeared with the cursor placed randomly in a central position (25% to the left and right of the midpoint).

#### Ratings of liking

Continuous visual analog scale ranging from “not at all” (left anchor) to “very much” (right anchor). Using the keyboard, participants were instructed to move the cursor along the continuum to express the degree of liking of exerted effort by solving the math problem. In each trial, the scale appeared with the cursor placed randomly in a central position (25% to the left and right of the midpoint).

#### Math self‐concept scale

This short scale captures the participant's self‐report of mathematical capabilities.^26^ Responses were made on six‐point scales containing endpoints of 1 (Strongly disagree) and 6 (Strongly agree) (*α* = 0.88).

### Procedure

**FIGURE 3:**
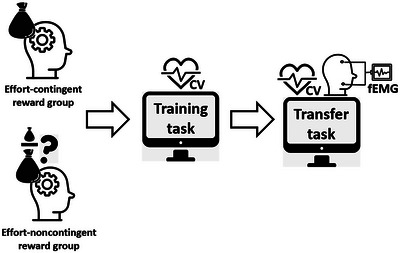
Illustration of the experimental procedure. Participants were first divided into the effort‐contingent and noncontingent (random) reward groups and then underwent a training task (*N*‐back) and a transfer task (MET) in a consecutive order. CV, cardiovascular measurements; fEMG, facial electromyography; MET, math effort task.

The experimental procedure is shown in Figure 3. First, participants filled in the consent forms and a set of questionnaires online from home. Upon coming to the lab, they were seated in front of the computer. The electrodes for CV measurements and the fEMG electrodes were applied. The experimenter then explained that the protocol includes an initial baseline period. During the baseline period, participants were to listen to a predetermined playlist selected for its affectively neutral content. After delivering baseline instructions, the experimenter returned to the control room to start a stopwatch and make baseline CV assessments. The CV response was recorded continuously during the 10‐min period. The baselines for each CV parameter were the mean of values obtained in the final 2 min. The CV responses were also continuously collected during both main tasks.

After the baseline period, the two main tasks followed. Participants were first randomly assigned to either the effort‐contingent or noncontingent reward group. The experiment started with the *N*‐back task (learning task) with 20 blocks consisting of 4 blocks of each 1‐, 2‐, 3‐, and 4‐back tasks in a randomized order (see Figure 1 and the *N*‐back task section). Participants were made aware that they will earn a reward after completing each block but were not instructed on what determines the reward. Following each block, the amount earned appeared on the screen. In the effort‐contingent group, unbeknownst to the participants, the exact amount of the reward received was contingent on participants’ effort mobilization as inferred from their PEP reactivity. This was determined by automatically subtracting the average PEP response obtained during the final 2 min of the baseline period from the average PEP responses obtained during each *N*‐back block. The difference between these two means determined the PEP reactivity score of each participant for each block and was utilized as the CV change score to determine reward. Participants received 1 cent for every 0.2 ms decrease in their PEP from baseline. Thus, a PEP that was 7.6 ms shorter during the *N*‐back block than it was at baseline would receive 38 cents as a reward for that block. In the noncontingent (random) reward group, participants had the reward amounts earned for each block yoked to the rewards gained by a previous participant in the effort‐contingent reward group. However, these rewards were distributed randomly amongst the 20 *N*‐back blocks. In doing so, we guaranteed that both groups received the same amount of reward with the only difference being if they were contingent on effort or not. Moreover, there were no initial group differences in effortful engagement during *N*‐back (*F*(1,192) = 0.69, *p* = 0.410). This implied that the groups were initially comparable in their levels of engagement.

Next, participants completed the modified MET (transfer task) (see Figure 2 and the MET section). Both CV and fEMG were continuously measured during this task. Upon completion of the whole experiment, participants were debriefed and got paid.

### Statistical analyses

To test the preregistered hypotheses, separate linear mixed model (LMM) analyses at the trial‐by‐trial level were fitted by muscle, with group (experimental/control) and difficulty (low/high) as fixed effects and by‐subject random intercepts and slopes for difficulty (unless the model did not converge or resulted in singular fits, in which case the random effects structure was simplified). However, models with random slopes frequently failed to converge or resulted in singular fits. Following best practices for model simplification,^27^ we retained by‐subject random intercepts to account for within‐subject dependencies while ensuring model stability. Math self‐concept was used as a covariate to control for participants’ preexisting differences in valuation of math. To test whether the removal of the random slope for difficulty might have increased Type I error rates, we conducted a null data simulation with 5000 iterations on the preregistered analyses that resulted in significant findings, where the dependent variable was replaced with randomly generated values from a normal distribution. We then refit our simplified model and tested whether it detected false positives. The results showed that Type I error rates for group (0.055), difficulty (0.052), and their interaction (0.052) were consistent with the nominal significance level of 0.05, indicating that Type I error rates were not inflated. We additionally also conducted parametric bootstrapping (5000 iterations) for both the maximal and the simplified model, and the bootstrapped standard errors for fixed effects were nearly identical across models (intercept: SE = 2.830 [simplified] versus 3.007 [maximal]; group: SE = 2.101 [simplified] versus 2.074 [maximal]; difficulty: SE = 2.020 [simplified] versus 2.017 [maximal]; interaction group × difficulty: SE = 2.989 [simplified] versus 2.942 [maximal]), further suggesting that removing the random slope did not meaningfully change uncertainty estimates or inflated Type I errors.

After detecting a significant interaction, we continued with simple effect (categorical predictors) or simple slopes (continuous predictors) analyses on the modeled data, decomposing the full interaction picture. In all analyses, we further controlled for the false discovery rate (FDR) associated with multiple testing using the Benjamini–Hochberg method.^28^ Note that the current analytical model is a slightly updated version of the original preregistration. We have now dropped the redundant subjective ratings factor from the muscle analyses as we treated subjective ratings also as dependent variables in other analyses. For better interpretability and because of an overall robustness of LMM analyses against non‐normality,^29^, ^30^ we kept the data untransformed. As with frequentist statistics it is not possible to provide evidence in favor of the null hypothesis (evidence of absence; see Ref. 31), any null findings on the sought interaction (group × difficulty) or the simple group effect were additionally followed up with Bayesian analyses in JASP 0.18.3. The default multivariate Cauchy prior (*r* scale prior width for fixed effects = 0.5) was used for the Bayesian ANCOVAs. The Bayes factors (BF01) were computed to estimate the evidence in favor of the null hypotheses, where BF01 between 3 and 10 is considered moderate, and a BF01 larger than 10 is considered strong evidence for the null hypothesis.^32^

## RESULTS

### fEMG analyses

First, we investigated the fEMG activity for each muscle at each phase (anticipation, task engagement, and post‐engagement).

#### Anticipation phase

There were no group differences for simple or interaction effects for both muscles (zygomaticus: all *F*s < 0.148, all FDR *p*s > 0.701; corrugator: all *F*s < 0.279, all FDR *p*s > 0.598; Tables S1 and S2). For the zygomaticus, the Bayes factor analysis provided very strong support for the absence of group × difficulty interaction (BF01 = 313.977) as well as for the simple group effect (BF01 = 14.155). For the corrugator activity, the Bayes factor analysis provided strong support for the absence of a group × difficulty interaction (BF01 = 350.406) as well as for the simple group effect (BF01 = 14.858).

#### Task engagement phase

Similarly, in the task engagement phase, there was no significant group effect or interaction (zygomaticus: all *F*s < 1.968, all FDR *p*s > 0.160; corrugator: all *F*s < 10.903, all FDR *p*s > 0.342; Tables S3 and S4). For the zygomaticus, the Bayes factor analysis provided very strong support for the absence of a group × difficulty interaction (BF01 = 123.752) as well as for the simple group effect (BF01 = 13.906). For the corrugator, the Bayes factor analysis provided strong support for the absence of a group × difficulty interaction (BF01 = 196.735) as well as for the simple group effect (BF01 = 14.053).

#### Post‐engagement phase

**FIGURE 4:**
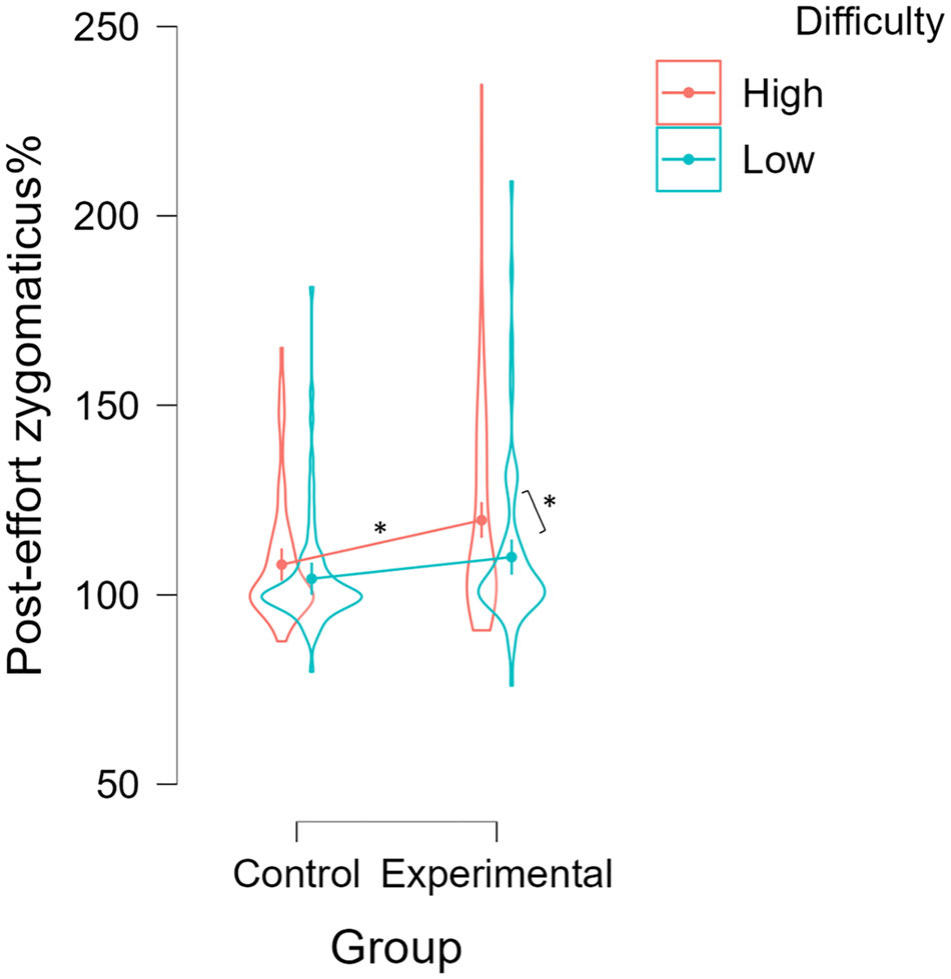
Illustration of the group × difficulty interaction for the post‐engagement zygomaticus major activity. Only the more difficult trials (red) led on average to greater zygomaticus reactivity, and only in the experimental but not in the control group. The figure represents model‐fitted zygomaticus data that are expressed as a % of the baseline and show average values for each group and difficulty along with their 95% confidence intervals and an overall data distribution (violin element), where each observation represents a single participant's average.

For the zygomaticus muscle, the results indicated a significant group × difficulty interaction (*F*(1,5,634.5) = 5.049, FDR *p* = 0.024; Table 1, Figure 4). The follow‐up simple effect analyses yielded support for our hypothesized relationship. Specifically, in the high difficulty trials there was greater zygomaticus activity in the experimental group (control—experimental; estimate = −11.7, SE = 3.12, *z* = −3.759, *p* < 0.001).^a^ In low difficulty trials, the difference between groups did not reach significance (control—experimental; estimate = −5.1, SE = 3.11, *z* = −1.640, *p* = 0.101). However, the follow‐up Bayes factor analysis provided anecdotal to moderate support for the presence of a meaningful difference between experimental and control group in the low difficulty (BF01 = 0.30), with the higher values in the experimental group. In the experimental group, high difficulty trials were characterized by significantly greater zygomaticus activity (high–low; estimate = 10.36, SE = 2.18, *z* = 4.763, *p* < 0.001). In the control group, the difference in zygomaticus activity between high and low difficulty trials was not significant (high–low; estimate = 3.73, SE = 1.99, *z* = 1.874, *p* = 0.066). The follow‐up Bayes factor analysis supported this result by providing only anecdotal evidence for a meaningful difference between high and low difficulty for the control group (BF01 = 0.558).

**TABLE 1:** Linear mixed model summary table for the post‐engagement zygomaticus analysis.

Variable	Sum Sq	Mean Sq	NumDF	DenDF	*F* value	*p*
Group	29,343	29,343	1	150.7	9.403	0.003
Difficulty	71,240	71,240	1	5634.5	22.828	<0.001
Math self‐concept	2488	2488	1	150.4	0.797	0.373
Group × Difficulty	15,756	15,756	1	5634.5	5.049	0.024

Abbreviations: DenDF, denominator degrees of freedom; NumDF, numerator degrees of freedom.

For the corrugator muscle, there was no significant group effect or interaction (all *F*s < 3.558, all FDR *p*s > 0.061; Table S5). The Bayes factor analysis provided strong support for the absence of a group × difficulty interaction (BF01 = 39.895) and moderate support for the absence of the simple group effect (BF01 = 3.485).

### Subjective ratings

To further assess whether the effort‐contingent reward in the experimental group led to a significant increase in subjective ratings in solving the high difficulty math problems, we performed separate LMMs, one for wanting and another for liking.

#### Wanting

Results did not show any group differences for simple or interaction effects (all *F*s < 0.048, all FDR *p*s > 0.828; Table S6). The Bayes factor analysis provided strong support for the absence of a group × difficulty interaction (BF01  = 13,188.61) as well as for the simple group effect (BF01 = 606.21).

#### Liking

Similarly, there were no group differences for simple or interaction effects (all *F*s < 3.433, all FDR *p*s > 0.065; Table S7). The Bayes factor analysis provided strong support for the absence of a group × difficulty interaction (BF01 = 23075.29) as well as for the simple group effect (BF01 = 5530.40).

**FIGURE 5:**
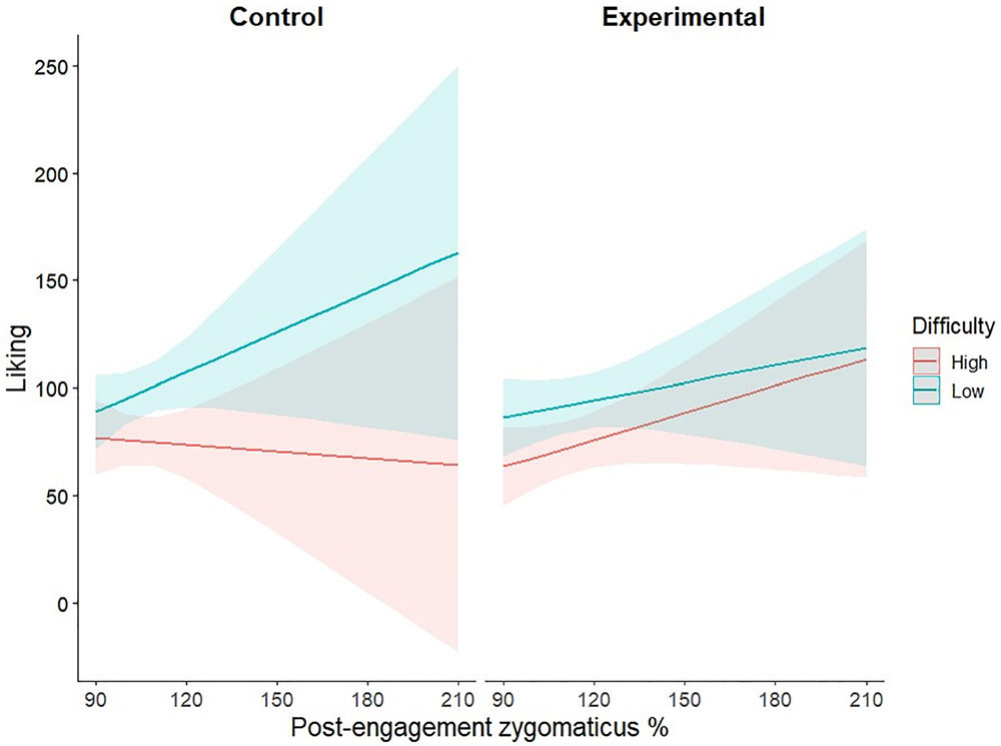
Illustration of the post‐engagement zygomaticus × group × difficulty interaction (*p* < 0.001) in predicting the liking ratings (*y*‐axis). The figure depicts a separate plot for the control group (left) and for the experimental group (right). Low difficulty trials are marked in blue and high difficulty trials are marked in red. In the difficult trials, the increasing zygomaticus engagement predicts the increase in liking, but only in the experimental group. In the low difficulty trials, the increasing zygomaticus engagement predicts liking to a lesser extent in the experimental (versus control) group.

As we previously found that specifically the post‐engagement zygomaticus activity was higher in the experimental versus control group, especially after the high difficulty trials, we used it as an additional predictor in a separate exploratory analysis of post‐engagement liking. Importantly, by doing that we also tested whether the increased zygomaticus activity in the post‐engagement phase was reflecting a mere contrast effect^33,34^ (i.e., greater pleasure in the experimental group caused by termination of adversity during effortful engagement). We found a significant group × difficulty × post‐engagement zygomaticus interaction (*F*(1,4,848.1) = 39.461, FDR *p* < 0.001; Table 2, Figure 5). To further inspect the nature of this three‐way interaction, we conducted a simple slopes analysis to compare the relationship between zygomaticus activity and liking ratings across the levels of group and difficulty. Pairwise comparisons of the slopes were performed and revealed that as opposed to low difficulty trials, in high difficulty trials, the increasing zygomaticus activity predicted increased liking ratings, and this was the case only in the experimental but not in the control group (control—experimental, high—low difficulty; estimate = −0.857, SE = 0.136, *z* = −6.282, *p* < 0.0001; Figure 2). This argues against the contrast hypothesis (pleasure caused by relief from pain), which would have predicted a negative association between post‐effort zygomaticus activity and liking. In our study, participants in the experimental group smiled more after difficult blocks when they had liked solving the math problems.

**TABLE 2:** Linear mixed model summary table for the liking analysis with the added predictor of post‐engagement zygomaticus both between (id) and within (cwc) subjects.

Variable	Sum Sq	Mean Sq	NumDF	DenDF	*F* value	*p*
Post‐engagement zygomaticus id mean	1816	1816	1	123	1.181	0.279
Post‐engagement zygomaticus cwc	1901	1901	1	4848	1.236	0.266
Group	3	3	1	123	0.002	0.965
Difficulty	2843	2843	1	4848.1	1.849	0.174
Math self‐concept	27,437	27,437	1	123	17.845	<0.001
Post‐engagement zygomaticus id mean × group	5	5	1	123	0.003	0.956
Post‐engagement zygomaticus cwc × group	3740	3740	1	4848	2.433	0.119
Post‐engagement zygomaticus id mean × difficulty	30,007	30,007	1	4848.1	19.516	<0.001
Post‐engagement zygomaticus cwc × difficulty	13	13	1	4852.5	0.009	0.926
Group × difficulty	53,565	53,565	1	4848.1	34.839	<0.001
Post‐engagement zygomaticus id mean × group × difficulty	60,673	60,673	1	4848.1	39.461	<0.001
Post‐engagement zygomaticus cwc × group × difficulty	3551	3551	1	4852.5	2.310	0.129

Abbreviations: DenDF, denominator degrees of freedom; NumDF, numerator degrees of freedom.

### Exploratory analyses

#### Performance

To address the potential alternative explanation that our findings were generated by group differences in performance (i.e., success in solving the math problems eliciting more pleasure), we checked the comparability of performance in the transfer task (MET; correct/false) between the experimental and the control group. We found no group effect or group × difficulty interaction (all *ps* > 0.638), and thus no support for this claim. We found only a significant main effect of difficulty with more difficult trials resulting in a lower chance of successful solving (OR = 0.21, *p* < 0.001; Table S8). We further included trial‐level performance as a predictor of post‐engagement zygomaticus activity and found no significant association (*F*(1,5,781.4) = 2.311 *p* = 0.128).

#### PEP

We explored whether the two groups were comparable in the amount of effort mobilization during the training task (*F*(1,192) = 0.69, *p* = 0.410) as well as during the transfer task (*F*(1,151.7) = 0.943, all FDR *p* = 0.333).

We also explored whether there was any interaction effect of group with difficulty, resulting in no significant interaction (*F*(1,6008) = 0.687, all FDR *p* = 0.407; Table S9). As expected, there was a strong effect of task difficulty with greater difficulty leading to greater task engagement (*F*(1,6008) = 56.644, all FDR *p* < 0.001). The Bayes factor analysis on the null results of experimental condition provided strong support for the absence of any exploratory group × difficulty interaction (BF01 = 35.462). At the same time, it provided only very weak support for the absence of the simple group effect (BF01 = 1.846).

We additionally tested the association of PEP and the d‐prime (the known behavioral measure of performance in the *N*‐back) that some attribute to possibly track subjective cognitive effort. We found no significant association between d‐prime and PEP ∆ (*rs* = −0.08, *p* = 0.268; note: the same was true also for each group separately, both *ps* > 0.368).

## DISCUSSION

The aim of this study was to investigate reward processing in participants who underwent a learned industriousness treatment to test whether they show signs of effort‐related experience of pleasure. We provided experimental evidence that rewarding participants for the exertion of cognitive effort can create hedonic value. Participants in the experimental group had greater hedonic reactions after working on the effortful transfer task and specifically after solving high difficulty math problems. This increased hedonic reaction was indicated by the relative increase in the ZM electrical muscle activity and further supported by the finding that this activity predicted subjective pleasure ratings (liking) in the experimental group. These effects were present despite a very short learning phase and are consistent with the assumption derived from learning theory that effort can gain the quality of a secondary reinforcer and become intrinsically rewarding when frequently paired with extrinsic reward.^5^

Stimuli eliciting pleasure typically trigger ZM activity,^35^ and this activity is specific for positively and not negatively valenced stimuli.^35^ This is to be expected, as the zygomaticus is primarily involved in smiling.^36^ In this study, we detected increased zygomaticus activity in the experimental group, but only after effort exertion. It thus indicates that pleasure was not evoked during effort exertion itself (task engagement phase) but appears as a result of working on cognitively difficult tasks. Such poststimulus reaction is still interpreted in the literature as consummatory pleasure (e.g., Refs. 21 and 37), as it reflects the momentary pleasure that is experienced immediately after engaging in an activity and is not linked to extrinsic reward.^38^ An explanation for this post‐effort pleasure could be that it mirrors the exact reward‐structure of the training phase. Participants received effort‐contingent reward only after they exerted effort, but not during effort exertion. The secondary reinforcer quality that effort gained as the result of effort‐contingent reward, and that presumably conveyed an intrinsic reward value to effort, is thus not elicited by the state of exerting high effort but rather by the state of having exerted high effort.

Our findings of the experimental group having higher zygomaticus activity after high difficulty trials could alternatively be explained by the so called contrast hypothesis.^33^, ^34^ Here, the higher post‐effort zygomaticus activity could be caused simply by termination of the experienced adversity of effortful task engagement. Specifically, according to the contrast hypothesis, higher effort in a task is aversive (similar to physical pain), which leads in turn to a greater positive affective shift when the task is completed (and effort is no longer required), compared to when a task requires less effort. As a result pleasure is experienced after the more effortful trials because they include a greater positive shift back to the equilibrium state once they are finished.^33^ If this was indeed the case for our data, one would expect that increased post‐effort zygomaticus activity would correlate with decreased subjective liking of such difficult trials. To the contrary, we found that increasing zygomaticus activity positively predicted self‐rated liking of the difficult trials in the experimental group. This pattern of results is more in line with the hypothesis of effort becoming a secondary reinforcer and gaining rewarding properties.

Although significantly less activated, we found numerically increased post‐effort zygomaticus activity also in the control group, namely, in high difficulty trials. There, we first ruled out the possibility of ZM tracking subjective pleasure because no correlation with explicit liking ratings was observed, which was different from the experimental group. Nevertheless, it is legitimate to speculate that a different process may drive the activity of the ZM during high difficulty in the control group. For example, in the study by Boxtel and Jessurun,^39^ it was found that toward the end and right after a very demanding cognitive task, zygomaticus activity increased. This was interpreted to occur in situations where suboptimal performance can no longer be sufficiently compensated by additional resources; therefore, ZM was possibly tracking feelings of distress rather than hedonic pleasure. Therefore, we tested for this alternative interpretation by performing a correlation analysis between ZM activity and effort. We did not find any association between effort (PEP) and the zygomaticus activity in any of the groups (all *ps* > 0.533). Given the lack of association with subjective experience or objective indicators, it is hard to interpret the increased ZM activity during difficult trials in the control group without falling in reverse inference, and we refrain from doing so.

We did not observe any increased zygomaticus activity occurring before task engagement, suggesting that participants did not experience pleasure from the anticipation of the future activity or simply did not anticipate it (anticipatory pleasure). The occurrence of anticipatory pleasure, a phenomenon possibly distinct from wanting or desire, is still under‐investigated, especially with objective measures such as fEMG. A few studies in humans have suggested that implicit hedonic experiences also occur during anticipation and can be modulated by dopaminergic and opioidergic activity.^13^, ^40^, ^41^ But whether anticipatory pleasure is always present and how much it differs from wanting is still a matter of investigation. Some insights come from a recent study demonstrating that reward cues may be liked initially and wanted later, due to an early affective evaluation of instructed expectations related to a task.^42^ This study, however, documented a higher evaluative effect, namely, for the explicit reward cues rather than for the cue instructions to task difficulty. In our experiment, the cue was also associated with task difficulty, but the association between the exerted effort and the reward was implicitly learned based on the exerted CV reactivity (PEP) in the experimental group. Such reward cue is therefore less salient (in accordance with Ref. 42) and thus may be less readily translated into altered conscious affective evaluations, or in our case, also into the related physiological reactions. Although it appears plausible that the anticipation of a reward usually elicits anticipatory pleasure, other evidence indicates that this is not always the case. Indeed, it has been shown that “wanting” (mediated by the mesolimbic dopamine system) is not always intrinsically pleasurable in itself, but under certain circumstances even negative or painful stimuli can gain incentive salience and become miswanted.^14^, ^43^ This shows that it is conceivable in principle that the desire to obtain an anticipated reward need not necessarily or always be accompanied by hedonic feelings of anticipatory pleasure but rather that the two types of responses can dissociate.

Different from our preregistered hypothesis and to the similar research of others,^44^ we did not find any group differences in the corrugator supercilii activity in any of the phases of the experiment. This result is possibly due to the fact that the effect might have been canceled out by the effortful nature of the task, since effortful tasks generally evoke greater contraction of the corrugator.^30^, ^31^ We explored and confirmed this by the positive correlation in the corrugator activity during effort with the effort‐related PEP (*r_s_* = 0.04, *p* < 0.001). In contrast, a recent study documented attenuation of the corrugator muscle in a cognitively effortful task when higher rewards were anticipated.^44^ Future studies should further address whether corrugator signaling is sensitive specifically in the learned industriousness scenarios.

One strength of our work is the use of sympathetic‐related CV reactivity (assessed as PEP) to realize effort‐contingent reward in the experimental group. Nearly four decades of research have established that this noninvasive psychophysiological measure is a reliable indicator of effort mobilization (for a review, see Ref. 45), superior to performance, which is typically dependent on task characteristics. Nevertheless, we also explored the relationship between task performance (d‐prime scores) and PEP during the training phase. Results do not show a significant association between the two measures. This lack of convergence is possibly caused by task complexity.^46^ Specifically, previous findings indicate a positive relationship between performance and effort during tasks of low complexity but a negative relationship for tasks of high complexity.^46^ Given that the *N*‐back task is considered a high‐complexity task,^47^ high effort may even undermine performance, resulting in the nonsignificant correlation observed in our study.

Our findings have important practical implications suggesting that effort can indeed be rewarding. The enjoyment of effort may, in turn, be beneficial for goal progress (e.g., in academic or work contexts).^48^, ^49^ Therefore, incorporating the here‐described rewarding mechanisms into the development of incentive cultures that focus on intraindividual growth and improvement and less on valuing only successful outcomes may be beneficial for nourishing individual´s enjoyment and motivation to work on effortful tasks with possible broad beneficial outcomes. Future studies should investigate the underlying neural and neurochemical mechanisms related to such rewarding effort exertion in order to characterize, for example, the causal nature of such processes (e.g., when the experience of pleasure is blocked, do I still look for effort?).

This study utilized a multi‐method approach with fEMG to test the hedonic experience when working on an effortful task. When assessing the different phases of reward processing, we did not see indications for increased reward anticipation or anticipatory pleasure. However, the results provide evidence for an enhanced consummatory pleasure reaction after effort was exerted, which was associated with the subjective liking experience. Contrary to the currently dominant view that mental effort is experienced as aversive and intrinsically costly, the present study provides the first evidence that effort can become intrinsically rewarding and elicit pleasure even in the absence of extrinsic reward. Further research is necessary to corroborate these findings, but it seems that humans might indeed not be destined to always avoid or suffer from effortful challenges they are confronted with in their daily lives. Instead, they may actually take pleasure from previous effortful engagement.

## AUTHOR CONTRIBUTIONS

Jakub Kraus, Veronika Job, Giorgia Silani, Christopher Mlynski, and Thomas Goschke developed the study concept. Jakub Kraus, Veronika Job, Giorgia Silani, and Christopher Mlynski designed research. Jakub Kraus and Franziska Hartmann performed research. Jakub Kraus, Christopher Mlynski, Georgia Clay, and Franziska Hartmann analyzed the data. Jakub Kraus and Veronika Job wrote the original manuscript. Giorgia Silani, Christopher Mlynski, and Thomas Goschke reviewed and edited it.

## COMPETING INTERESTS

The authors declare no conflicts of interest.

### PEER REVIEW

The peer review history for this article is available at: https://publons.com/publon/10.1111/nyas.15323

## Supporting information




**Table S1**. Linear mixed model summary table for the anticipation zygomaticus analysis.
**Table S2**. Linear mixed model summary table for the anticipation corrugator analysis.
**Table S3**. Linear mixed model summary table for the task engagement zygomaticus analysis.
**Table S4**. Linear mixed model summary table for the task engagement corrugator analysis.
**Table S5**. Linear mixed model summary table for the post‐engagement corrugator analysis.
**Table S6**. Linear mixed model summary table for the wanting analysis.
**Table S7**. Linear mixed model summary table for the liking analysis.
**Table S8**. Linear mixed model summary table for the performance analysis.
**Table S9**. Linear mixed model summary table for the PEP analysis.

## Data Availability

The data that support the findings of this study are openly available in OSF at https://doi.org/1017605/OSF.IO/N2T3J.

## Footnotes

a The use of *z*‐statistics is specific to post hoc pairwise comparisons and is based on the Wald test (coefficient divided by standard error). It is utilized for larger samples where the *z*‐distribution closely approximates the *t*‐distribution and is consistent with standard practices in mixed‐effects modeling, particularly when reporting simple effects or pairwise comparisons derived from the model.
