# Supplementary material for: The pleasure of effort: Cognitive challenges trigger hedonic physiological responses
Source: Ann N Y Acad Sci. 2025 Mar 24;1546(1):100–11. doi: 10.1111/nyas.15323 (PMC11998477; doi:10.1111/nyas.15323)
Supplement: Supplementary file 1 — Table S1. Linear mixed model summary table for the anticipation zygomaticus analysis. Table S2. Linear mixed model summary table for the anticipation corrugator analysis. Table S3. Linear mixed model summary table for the task engagement zygomaticus analysis. Table S4. Linear mixed model summary table for the task engagement corrugator analysis. Table S5. Linear mixed model summary table for the post‐engagement corrugator analysis. Table S6. Linear mixed model summary table for the wanting analysis. Table S7. Linear mixed model summary table for the liking analysis. Table S8. Linear mixed model summary table for the performance analysis. Table S9. Linear mixed model summary table for the PEP analysis. [file NYAS-1546-100-s001.docx]

**Supporting Material**

**Supporting Table S1.** Linear mixed model summary table for the Anticipation zygomaticus analysis.

| **Variable** | **Sum Sq** | **Mean Sq** | **NumDF** | **DenDF** | **F value** | **P** |
| --- | --- | --- | --- | --- | --- | --- |
| Group | 228 | 228 | 1 | 149 | 0.148 | 0.701 |
| Difficulty | 2662.6 | 2662.6 | 1 | 5589.6 | 1.725 | 0.189 |
| Math self-concept | 3474.3 | 3474.3 | 1 | 149.2 | 2.251 | 0.136 |
| Group*Difficulty | 220.4 | 220.4 | 1 | 5589.6 | 0.143 | 0.706 |

**Supporting Table S2.** Linear mixed model summary table for the Anticipation corrugator analysis.

| **Variable** | **Sum Sq** | **Mean Sq** | **NumDF** | **DenDF** | **F value** | **P** |
| --- | --- | --- | --- | --- | --- | --- |
| Group | 121.37 | 121.37 | 1 | 152.3 | 0.279 | 0.598 |
| Difficulty | 488.28 | 488.28 | 1 | 5734.1 | 1.121 | 0.290 |
| Math self-concept | 136.17 | 136.17 | 1 | 152.4 | 0.313 | 0.577 |
| Group*Difficulty | 2.16 | 2.16 | 1 | 5734.1 | 0.005 | 0.944 |

**Supporting Table S3.** Linear mixed model summary table for the Task engagement zygomaticus analysis.

| **Variable** | **Sum Sq** | **Mean Sq** | **NumDF** | **DenDF** | **F value** | **P** |
| --- | --- | --- | --- | --- | --- | --- |
| Group | 909.7 | 909.7 | 1 | 149.5 | 0.789 | 0.376 |
| Difficulty | 0.9 | 0.9 | 1 | 5631.2 | 0.001 | 0.978 |
| Math self-concept | 945.66 | 945.66 | 1 | 149.3 | 0.820 | 0.367 |
| Group*Difficulty | 2269.57 | 2269.57 | 1 | 5631.2 | 1.968 | 0.161 |

**Supporting Table S4.** Linear mixed model summary table for the Task engagement corrugator analysis.

| **Variable** | **Sum Sq** | **Mean Sq** | **NumDF** | **DenDF** | **F value** | **P** |
| --- | --- | --- | --- | --- | --- | --- |
| Group | 3.06 | 3.06 | 1 | 152.8 | 0.005 | 0.941 |
| Difficulty | 1749.26 | 1749.26 | 1 | 5757.3 | 3.087 | 0.079 |
| Math self-concept | 775.54 | 775.54 | 1 | 152.9 | 1.369 | 0.244 |
| Group*Difficulty | 511.55 | 511.55 | 1 | 5757.3 | 0.903 | 0.342 |

**Supporting Table S5.** Linear mixed model summary table for the Post-engagement corrugator analysis.

| **Variable** | **Sum Sq** | **Mean Sq** | **NumDF** | **DenDF** | **F value** | **P** |
| --- | --- | --- | --- | --- | --- | --- |
| Group | 3054.7 | 3054.7 | 1 | 153 | 3.559 | 0.061 |
| Difficulty | 6432.8 | 6432.8 | 1 | 5757.9 | 7.494 | 0.006 |
| Math self-concept | 690.4 | 690.4 | 1 | 153 | 0.804 | 0.371 |
| Group*Difficulty | 1250.2 | 1250.2 | 1 | 5757.9 | 1.457 | 0.228 |

**Supporting Table S6.** Linear mixed model summary table for the Wanting analysis.

| **Variable** | **Sum Sq** | **Mean Sq** | **NumDF** | **DenDF** | **F value** | **P** |
| --- | --- | --- | --- | --- | --- | --- |
| Group | 72 | 72 | 1 | 132 | 0.050 | 0.830 |
| Difficulty | 224022 | 224022 | 1 | 5127.1 | 147.070 | <0.001 |
| Math self-concept | 23373 | 23373 | 1 | 132 | 15.340 | <0.001 |
| Group*Difficulty | 10 | 10 | 1 | 5127.1 | 0.010 | 0.930 |

**Supporting Table S7.** Linear mixed model summary table for the Liking analysis.

| **Variable** | **Sum Sq** | **Mean Sq** | **NumDF** | **DenDF** | **F value** | **P** |
| --- | --- | --- | --- | --- | --- | --- |
| Group | 15 | 15 | 1 | 132 | 0.010 | 0.921 |
| Difficulty | 626113 | 626113 | 1 | 5262 | 411.689 | <0.001 |
| Math self-concept | 30832 | 30832 | 1 | 132 | 20.273 | <0.001 |
| Group*Difficulty | 5221 | 5221 | 1 | 5262 | 3.433 | 0.064 |

**Supporting Table S8.** Linear mixed model summary table for the Performance analysis

| **Variable** | **Odds Ratios** | **CI** | **Statistic** | **p** |
| --- | --- | --- | --- | --- |
| Group | 1.01 | 0.78 – 1.32 | 0.10 | 0.922 |
| Difficulty | 23.75 | 18.92 – 29.82 | 27.28 | <0.001 |
| Math self-concept | 1.14 | 1.02 – 1.27 | 2.39 | 0.017 |
| Group*Difficulty | 0.92 | 0.66 – 1.28 | -0.47 | 0.638 |

**Supporting Table S9.** Linear mixed model summary table for the PEP analysis.

| **Variable** | **Sum Sq** | **Mean Sq** | **NumDF** | **DenDF** | **F value** | **P** |
| --- | --- | --- | --- | --- | --- | --- |
| Group | 13.31 | 13.31 | 1 | 151.7 | 0.944 | 0.333 |
| Difficulty | 798.96 | 798.96 | 1 | 6008.7 | 56.644 | <0.001 |
| Math self-concept | 30.23 | 30.23 | 1 | 151.7 | 2.144 | 0.145 |
| Group*Difficulty | 9.69 | 9.69 | 1 | 6008.7 | 0.687 | 0.407 |
